# Anticaries Agent Based on Silver Nanoparticles and Fluoride: Characterization and Biological and Remineralizing Effects—An In Vitro Study

**DOI:** 10.1155/2022/9483589

**Published:** 2022-04-19

**Authors:** Jaqueline Costa Favaro, Tiago Roberto Detomini, Luciana Prado Maia, Regina Célia Poli, Ricardo Danil Guiraldo, Murilo Baena Lopes, Sandrine Bittencourt Berger

**Affiliations:** ^1^Department of Restorative Dentistry, University of North Parana, Department of Restorative Dentistry, Rua Marselha, 183, Londrina 86.041-140, PR, Brazil; ^2^Department of P&D, Biodinâmica Química e Farmacêutica, Rua Ronat Valter Sodré, 4350, Ibiporã 86200-000, PR, Brazil

## Abstract

**Objective:**

The aim of the study was to characterize and evaluate the stability, antimicrobial activity, cytotoxicity, and remineralizing effects of silver nanoparticles and fluoride anticaries agent (AgF) on staining dental enamel.

**Materials and Methods:**

An experimental AgF solution was prepared and compared to silver diamine fluoride (SDF). First, the AgF was characterized and the stability was evaluated by transmission electron microscopy (TEM). *Streptococcus mutans*, *Enterococcus faecalis*, and *Escherichia coli* strains were used to evaluate the minimum inhibitory and bactericidal concentration and cytotoxicity performed using L929 fibroblastic cells by MTT test. Caries-like lesions induced by pH-cycling in human enamel were obtained, and then, the superficial microhardness, cross-sectional microhardness (CSMH), scanning electron microscopy (SEM), and energy-dispersive X-ray spectroscopy (EDS) were performed. Photographic images were taken to analyze the enamel staining.

**Results:**

The AgF showed stableness in long term with bacteriostatic and bactericidal actions without cytotoxicity. Enamel remineralization, in surface and in depth (CSMH), was observed when the AgF was used, and it was similar to SDF. SEM showed enamel precipitation, and EDS observed the presence of P, Ca, Au, Ag, and Cl elements. Contrary to SDF, AgF did not stain the enamel.

**Conclusion:**

The nano silver fluoride anticaries agent tested presented long-term stability, superficial and in-depth remineralizing capacity with antimicrobial potential and biocompatibility and did not stain the enamel.

## 1. Introduction

Topical anticaries agents provide a noninvasive method for controlling and preventing dental caries. Silver diamine fluoride (SDF) is a colorless liquid based on ammonia hydroxide, silver nitrate, and hydrofluoric acid, which can paralyze the caries progression without removing the lesion, and its usage has been documented since 1969 [1]. In August 2014, SDF was cleared by the Food and Drug Administration for hypersensitivity treatment in adults over 21 years old [2, 3]. Inhibiting demineralization and promoting remineralization are the possible action mechanisms for stopping caries [4, 5], which are associated with silver nitrate for antibacterial effect [6]. However, the SDF blackened the areas affected by the carious process; this is due to the process of reducing the silver ion contained in its formulation [7–10], making this an antiaesthetic material. Thus, despite the high success rate [8], this darkening effect restricts its usage [8, 9].

Silver nanoparticles are very effective antimicrobial agents, whose action is greater when compared with ionic silver [9]. They have a greater surface area available to interact with the microorganism, and their antibacterial activity is related to the size of its particle; that is, its antimicrobial effect increases with the decrease of particle size [2, 11–13]. The antimicrobial mechanism of silver nanoparticles occurs because of their ability to penetrate the bacterial cell wall, damaging it by direct and indirect lipid peroxidation, thus interrupting cell processes such as replication of DNA and inhibition of cellular respiration [2, 9, 14].

The development of new anticaries agents with the presence of silver nanoparticles in the composition could eliminate the aesthetic damage caused by the dental surface pigmentation when SDF is used [8, 9]. Moreover, the antibacterial and remineralizing properties [2, 8, 9, 14–17] with low or no cytotoxicity [2, 9] can be achieved. However, for the effectiveness and to be the replacement of ionic silver in anticaries solutions, these silver nanoparticles should remain stabilized in a colloidal solution so that there will be no constant interaction with bacteria [13, 16]. Also, the presence of fluoride is necessary; this is because when fluoride is associated with silver nanoparticles, it gives a synergistic effect that promotes the remineralization of the tooth enamel [4, 8, 14] and bactericidal action against cariogenic microorganisms [8].

The possibility of making a minimally invasive dental caries treatment without the formation of aerosol that is available to dental professionals, which can preserve the healthy dental structure, promote remineralization, and prevent the progression of the disease without darkening the dental surface, will bring new perspectives for controlling and treating caries and then preventing the spread of microorganisms through aerosol. Given the absence of studies that demonstrate the stability of a cariostatic solution with the presence of silver and fluorine nanoparticles in the composition to make it clinically applicable, the objective of this study was to evaluate the effects of an experimental anticaries agent based on silver nanoparticles and fluoride (AgF) on human dental enamel, its potential antimicrobial action, degree of cytotoxicity, the stability of nanoparticles in the solution in the short term and long term (12 months), and enamel staining. The null hypothesis was that there is no difference in the remineralization and antimicrobial effects between the anticaries agents tested (SDF and AgF).

## 2. Materials and Methods

The study was approved by the Research Ethics Committee of University of North Parana (protocol # 2.531.210). In this study, a commercially available anticaries agent, SDF (Cariestop 30%; Biodinâmica, Ibiporã, Parana, Brazil), and an experimental solution (AgF) that is under patent deposit in Brazil (protocol no. NBR102019024385-6) were tested. This one was based on silver nanoparticles and fluoride stabilized with ethylene glycol and polyvinylpyrrolidone (surfactant). For all analyses, the evaluator was blinded; however, due to the solutions presentation form, it was not possible to blind the operator.

### 2.1. Experimental Solution Description

The AgF is a two-component solution due to a pilot study that found that for long-term stabilization of the solution, the silver and fluoride nanoparticles need to be in separate bottles. In this way, two liquid compounds (parts A and B) were stored in separate bottles and mixed in a 1 : 1 ratio, at the time of use.

- Part A: colloidal silver nanoparticles solution with spherical particle an average 30 nm size at 0.04% stabilized with ethylene glycol and polyvinylpyrrolidone and water as a vehicle. This solution was obtained through chemical coprecipitation reactions with pH from 3 to 5.
- Part B: 2% sodium fluoride and water as vehicle with a pH between 6 and 8.

To verify the individual action of silver nanoparticles and fluoride, the experimental solution with only silver nanoparticles (Part A-Ag) and only fluoride (Part B-F) was used.

### 2.2. Characterization and Stability of Silver Nanoparticles

Immediately and after 12 months of experimental AgF preparation, the solution was evaluated using TEM (JEOL JEM-1400; Shimadzu Co., Kyoto, Japan) with an accelerating voltage of 120 kV operating at 80 kV. For the sample preparation, 5 *μ*L of an aqueous dispersion of each sample was deposited on a 200-mesh holey carbon film supported on a copper grid and dried at room temperature. TEM was used to obtain representative images of the AgF, to characterize the silver nanoparticles, and to demonstrate their stability in the colloidal solution, which will be demonstrated by their dispersion.

### 2.3. Antimicrobial Tests

#### 2.3.1. Minimum Inhibitory Concentration (MIC) and Minimum Bactericidal Concentration (MBC)

Assays were based on the criteria described by the National Committee for Clinical Laboratory Standards for bacteria, M07-A10 [18]. All the tests were performed in triplicate. *Streptococcus mutans* American Type Culture Collection (ATCC) 35668, *Enterococcus faecalis* ATCC 29212, and *Escherichia coli* ATCC 25922 were used. The microorganism strains were cultivated on brain heart infusion (BHI), and they were grown for 12 h in a liquid BHI at 37°C with agitation at 150 rpm. Suspensions of each microorganism were then adjusted to 0.5 McFarland in phosphate-buffered saline (PBS).

MICs of the experimental solutions, SDF, and turbidity were assessed by the spectrophotometric microdilution method, and we used chlorhexidine (CHX) as a positive control. All the wells were filled with the oxidation-reduction indicator resazurin (R7017; Sigma-Aldrich Inc., Darmstadt, Germany) to confirm the antimicrobial activity. Resazurin indicated the absence of microorganisms by blue (inhibition) or the presence of them by pink. Serial dilutions (1 : 2, 50 *μ*L) and CHX were inserted into the wells of a U-bottom 96-well plate. The concentrations of the tested solution in the first well were 500, 200, and 1200 *μ*g/mL for AgF, SDF, and CHX, respectively. The formulations with fluoride only (F) and with silver nanoparticles (Ag) were also incubated. Then, 50 *μ*L bacteria suspension was added to each well. We used the wells with microorganisms without tested solutions as a negative control. The absorbance of each well was determined on an enzyme-linked immunosorbent assay microplate reader (KHB ST-360; Shanghai Kehua Laboratory System Co., Ltd., Shanghai, China), adjusted to 630 nm, before and after incubation, for 24 h (for *E. faecalis* and *E. coli*) or 48 h (for *S. mutans*) at 37°C.

The MICs were defined as the lowest concentration capable to inhibit bacterial growth. The MBCs were defined as the concentrations at which MIC aliquots did not exhibit visible bacterial growth on agar plates [9].

#### 2.3.2. Agar Diffusion Test (ADT)

By using sterile cotton swabs, we spread the bacterial suspensions evenly across the surfaces of plates with solid BHI. Then, a 50 *μ*L solution was added each in three agar plate wells; for SDF, the adjusted concentration was 300 mg/mL; for AgF, we tested the concentration of 160 *μ*g/mL, defined from MIC and MBC results, and 1200 *μ*g/mL for CHX. The plates with *S. mutans* were incubated for 24 and 48 h at 37°C with 5% CO_2_, whereas plates with *E. coli* and *E. faecalis* were incubated without CO_2_. After incubation, the plates were visually analyzed, and the inhibition halos were measured with a digital caliper. The inhibition zone was calculated from (*a*) the ratio between the diameter of the halos and (*b*) the well in which the tested solutions were inserted. The absence of microbial activity was indicated by the value 1.0 (*a* = *b*).

#### 2.3.3. Cytotoxicity Assay with Quantitative Cell Viability Measurement

We used the culture of L929 fibroblasts because of the formation of dense tissue and the representation of a preestablished cell type in the study of cytotoxicity, according to classification and standards of ISO 10993 [19].

L929 cells (mouse conjunctive tissue, ATCC) (Adolfo Lutz Institute, São Paulo, São Paulo, Brazil) were seeded in 96-well plates (1 × 10^4^ cells/well) and kept in Eagle's minimum essential medium (Gibco, Glasgow, UK), supplemented with 10% of fetal bovine serum (Gibco) and 1% of antibiotic and antimycotic (Gibco). The cells were maintained in a humidified environment at 37°C with 5% CO_2_ and 95% atmospheric air for 24 h to form a semiconfluent monolayer. Then, cells were exposed to the extracts of the experimental solutions (SDF, AgF, F, and CHX) and CHX at 0.1% for 24 h. Untreated cells were used as a negative control. After this period, MTT (3-[4,5-dimethylthiazol-2yl]-2,5-diphenyltetrazolium bromide) (Sigma-Aldrich Inc.) solution (5 mg/mL) was added to the cultured medium at 10%, and the cells were maintained in a humidified environment at 37°C with 5% CO_2_ and 95% atmospheric air for 4 h. The solution was removed, and a dimethyl sulfoxide solution (Synth, Diadema, São Paulo, Brazil) was added (100 *μ*L/well). The plates were shaken for 5 min. Absorbance was detected at 570 nm (*μ*QuantTM; BioTek, Winooski, VT, USA). Each experiment was performed using three wells for each group and was repeated three times. The results were expressed as viability values relative to the negative control, using the equation:

(1) $$\begin{array}{c} \frac{\left[\left({\text{OD}}_{T}-{\text{OD}}_{B}\right)\times \left({\text{OD}}_{C}-{\text{OD}}_{B}\right)\right]}{100}, \end{array}$$

where OD means optical density, *T* means treatment, *B* means blank, and *C* means control.

### 2.4. Remineralization Analysis

#### 2.4.1. Sample Size

The sample size of 12 enamel blocks per experimental group was calculated based on a pilot study, considering the surface microhardness as the primary outcome (recovery of microhardness from demineralized enamel after the treatments). A microhardness recovery difference of 80 KHN with a standard deviation of 42 KHN, an *α*-error of 0.05, and a power (1 − *β*) of 0.9 were used.

#### 2.4.2. Specimens Preparation

In this study, specimens of intact enamel from human teeth were used. The specimens were prepared from enamel fragments obtained from the buccal and lingual crown surfaces of each tooth (4 mm × 4 mm × 3 mm) and inserted into a PVC pipe ring (0.75–1.5 cm) (Tigre, Castro, Parana, Brazil) with colorless acrylic resin (Jet Classico, São Paulo, Brazil).

**Figure 1:**
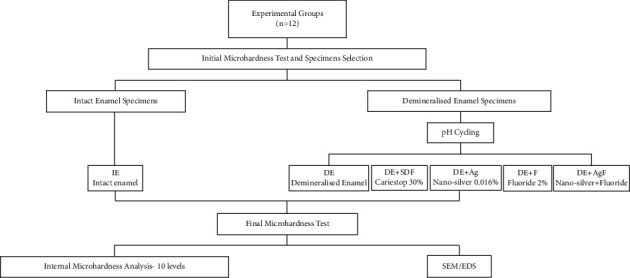
Flowchart of enamel remineralization analysis: scanning electron microscopy (SEM) and energy-dispersive X-ray spectroscopy (EDS).

Enamel surfaces were flattened using a series of silicon carbide papers (SiC) (600, 1200, and 2000 grit; 3M ESPE, Sumaré, São Paulo, Brazil) on the electric polishing machine (APL4; Arotec, Cotia, São Paulo, Brazil), at low rotation under running water to flatten the enamel. Afterward, the samples were polished with the felt disc (Arotec) and diamond paste in 1 and 0.25 *μ*m granulations (Arotec). Then, the samples were bathed with ultrasonicated distilled water (ultrasonic cleaner; Odontobras, Ribeirão Preto, São Paulo, Brazil) for 10 min and examined in a stereoscopic loupe (Bel Microimage Analyzer; Bel Photonics, Monza, Milan, Italy) to check for the absence of cracks and other enamel defects. Specimens were enumerated, isolated with nail enamel (Revlon, New York, NY, USA) to delimit a 7 mm^2^ exposure area of tooth enamel and stored in moisture to prevent dehydration.  Figure 1 shows the study design concerning different treatments for enamel remineralization after treatments. One group of specimens remained intact, whereas early caries lesions were formed in the other specimens.

#### 2.4.3. Surface Microhardness (SM) Test

Specimens' enamel surface microhardness was performed using a microdurometer (HMV-G 21S; Shimadzu Co.). For the reading, a Knoop (KHN) pyramidal diamond penetrator (HMV-G; Shimadzu Co.) was used with a static load of 50 gr per 10 s [20]. In each sample, three indentations were performed (100 *μ*m distance each one), and the mean values of the three indentations represented the value of the sample in the baseline.

#### 2.4.4. Specimens Selection and Division of Experimental Groups

The initial KHN values (SM_*i*_) made the specimen's selection. The overall mean KHN was calculated, and samples with values below or above 10% of the average were excluded from the study. The remaining were randomly divided into 6 experimental groups (*n* = 12), according to Figure 1. The specimens were then numbered and randomly allocated in acrylic matrices, according to a list generated according to the website (https://www.random.org/). To verify whether samples were statistically equivalent at baseline (*p* = 0.49), we performed one-way ANOVA.

#### 2.4.5. pH Cycling

To obtain the initial artificial caries lesion *in vitro*, the samples were submitted to pH cycling at 37°C for 8 days. The specimens were immersed for 8 h in the demineralizing solution (1.4 mM Ca, 0.9 mM P, 0.05 M acetate buffer, pH 5; 0.2 mL per specimen) and 16 h in the remineralizing solution (0.5 mM Ca, 0.9 mM P, 0.1 M tris buffer, pH 7; 2 mL per sample) [21]. The enamel SM was performed on a sample from each group daily until the obtained microhardness value was close to KHN = 150 [22]. Then, all specimens were again SM analyzed to verify enamel demineralization (SM_pH_). The average of each one was calculated, and one-way ANOVA was performed; the standardized demineralization of samples was verified (*p* = 0.1669).

#### 2.4.6. Experimental Treatment

The specimens were cleaned with pumice and Robinson's brush, washed, and dried, then received the application of the solution (according to group division shown in Figure 1) with a disposable applicator for 3 min. After 24 h, the SM of specimens were again analyzed, and the average of each sample was calculated to obtain final SM (SM_*f*_). The SM_*i*_, SM_pH_, and SM_*f*_ values were used to calculate the percentage change in superficial microhardness (%SM) by the following [23]:

(2) $$\begin{array}{c} \%\text{SM}=\left(\frac{{\text{SM}}_{f}-{\text{SM}}_{\text{pH}}}{{\text{SM}}_{i}-{\text{SM}}_{\text{pH}}}\right)\times 100. \end{array}$$

Further, the microhardness difference (ΔSM) was obtained by the following:

(3) $$\begin{array}{c} \Delta \text{SM}={\text{SM}}_{f}-{\text{SM}}_{\text{pH}}. \end{array}$$

#### 2.4.7. Cross-Sectional Microhardness (CSMH)

To evaluate the effect of solutions in dental enamel at depth, the CSMH readings were performed. For this, the samples were sectioned on their long axis with precision cutter diamond disc (Isomet 1000; Buehler, Lake Bluff, IL, USA). One half was used for CSMH measurement, and the other half was analyzed by scanning electron microscope (SEM). The specimens used for CSMH were embedded in a PVC tube ring (40 mm in diameter and 1.5 cm in height) (Tigre) with acrylic resin (Jet Classico), and the inner surfaces were polished the same as the surface enamel. The CSMH of each sample was measured by three impressions at depths of 20, 40, 60, 80, 100, 120, 140, 160, 180, and 200 *μ*m. The data were analyzed and submitted to the determination of the percentage of mineral volume (%MV), using the following [24]:

(4) $$\begin{array}{c} \%\text{MV}=4.3\left(\text{KHN}\right)\frac{1}{2}+11.3. \end{array}$$

#### 2.4.8. SEM and Energy-Dispersive X-Ray Spectroscopy (EDS)

The other half of the specimens was observed by SEM to surface morphological characterization. For this, the specimens were stored in a drying oven for 12 h, fixed in aluminum stubs with the aid of a double-sided carbon tape (Electron Microscopy Sciences, Washington, PA, USA), and coated with gold/palladium alloy on evaporator equipment (Balzers SCD 050 sputter coater, Balzers Union Aktiengesellschaft, and Fürstentum FL-9496; Balzers, Liechtenstein, Germany) by the 45 mA metallization process for 160 s. Specimens were analyzed on an SEM (Quanta 250; FEI, Hillsboro, OR, USA) at a voltage acceleration of 15 kV, 12 mm (work distance), and 20 nm spot-size lens aperture at 15,000× magnification.

In addition to SEM analysis, specimen chemical analyses were performed by EDS (EDS 6070; LEO Electron Microscopy/Oxford Microscopy, Cambridge, England). Thus, the qualitative microanalysis of the chemical elements might be present in each specimen and the chemical mapping of the dental surfaces.

#### 2.4.9. Photographic Images

To observe the superficial and cross-sectional enamel staining, we photographed the specimens from the SDF and AgF groups before and after 4-week treatments using a digital camera (EOS 70D, Canon, Tokyo, Japan), EF 100 mm 1 : 2.8 Macro USM Lens (Canon), and Macro Ring Lite Flash MR-14EX II (Canon). Standardized parameters were used for image acquisition, and the photographs were obtained at the same time of day. A 40-cm distance between the lens and samples was established.

### 2.5. Data Analysis

Data were analyzed using Minitab 19.1 for Windows 8 software (Minitab, Pennsylvania State College, Philadelphia, PA, USA). To evaluate the normality of data, we adopted the Kolmogorov–Smirnov test. The ADT data did not present normal distribution and were submitted to the Kruskal–Wallis test, followed by Dunn's test for comparison between groups. Surface microhardness and cytotoxicity assay presented normality and were submitted to one-way ANOVA (cytotoxicity, %SM, and ΔSM) and two-way ANOVA (SM), followed by Tukey's test. SEM and TEM images were qualitatively evaluated as well as the data of CSMH that were qualitatively analyzed along the depths and compared between the treatments. The level of 5% of significance was adopted.

## 3. Results

### 3.1. Characterization and Stability of Silver Nanoparticles

**Figure 2:**
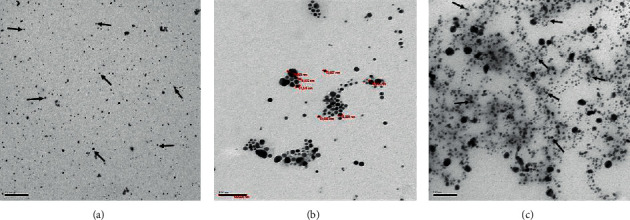
TEM micrograph of AgF solution immediately after manufacture; (a) predominantly spherical silver nanoparticles (arrow) dispersed in the colloidal solution; (b) higher magnification allows verifying the sizes of 7–30 nm of silver nanoparticles; (c) micrograph of solution 12 months after manufacture. The silver nanoparticles (arrow) remained dispersed in the solution after approximately 12 months of preparation (image with 100 nm scale).

Figure 2 shows the characterization of silver nanoparticles dispersed in the colloidal solution immediately and 12 months after solution preparation. The TEM analysis revealed the silver nanoparticles with a predominantly spherical shape and size of 7–30 nm dispersed in the solution.

### 3.2. Antimicrobial and Cytotoxic Effect

AgF, silver nanoparticles and fluoride 0.016%, was able to inactivate 100% growth of *S. mutans*, *E. faecalis*, and *E. coli* at a lower concentration of active compound than 30% SDF and 0.12% CHX digluconate (a positive control). The MIC and MBC values of the ATCC strains were 160 *μ*g/mL, and there was no difference between the MIC and the MBC values.

Table 1 presents the ADT results. All solutions presented inhibition halos; the absence of microbial activity is indicated by the value 1.0 (*a* = *b*), so values equal to or greater than 1 indicate that the solution had antimicrobial activity. For *S. mutans* and *E. faecalis*, SDF showed the largest inhibition zone, followed by CHX that was larger than AgF and Ag. For *E. coli*, SDF presented a larger zone and was statistically different from the other solutions tested.

**Figure 3:**
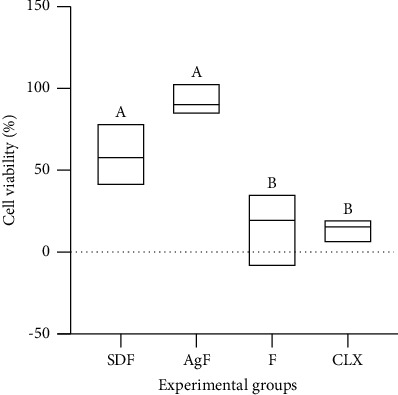
Cell viability percentage in relation to the control group. Different letters indicate a significant difference in intergroup comparisons.

Regarding cytotoxicity, AgF and SDF presented similar values, whereas F and CHX significantly reduced cell viability (*p* < 0.001) (Figure 3).

### 3.3. Enamel Alteration-Demineralization and Remineralization

#### 3.3.1. Surface Microhardness Alterations

In addition to the statistical tests described in Section 2.5, the power test of the percentage of surface microhardness (%SM) alteration was calculated using one-way ANOVA, which resulted in 99%, considering *α* = 0.05, the standard deviation of 34.59, and the number of levels equal to 6 (specified groups), with a maximum difference of 84.57 between groups *n* = 12. Only data from the six groups that were subjected to pH cycling received the application of anticaries agents because the other groups did not significantly change the %SM.

Table 2 shows the mean values and the standard deviation of the %SM, ΔSM, and assessment time (SH_*i*_, SH_pH_, and SH_*f*_). The ANOVA of %SM and ΔSH identified differences between the experimental groups (*p* < 0.001). For both (%SM and ΔSH), the groups treated with the SDF and AgF were statistically superior to F and Ag. Furthermore, when the assessment time was evaluated, ANOVA also identified statistical differences for the following factors: treatment (*p* < 0.001) and assessment time (*p* < 0.001). In addition, there was interaction between the factors (*p* < 0.001). In general, SM_*i*_ > SM_pH_ < SM_*f*_; however, in SH_*f*_, the groups treated with SDF and AgF showed values of microhardness statistically higher than the other groups.

#### 3.3.2. CSMH

**Figure 4:**
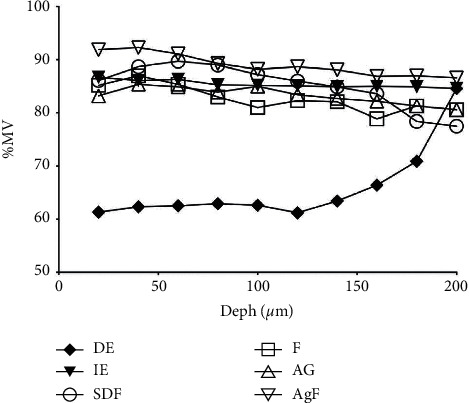
The percentage representation of mineral volume (%MV) in depths according to experimental groups.

Figure 4 shows the percentage of mineral volume (%MV) in depths according to experimental groups, and it is observed that DE group presented lower mineral volume in all depths. However, the treatments have the same pattern of mineral volume throughout the depths, and these were similar to intact enamel.

#### 3.3.3. SEM and EDS

**Figure 5:**
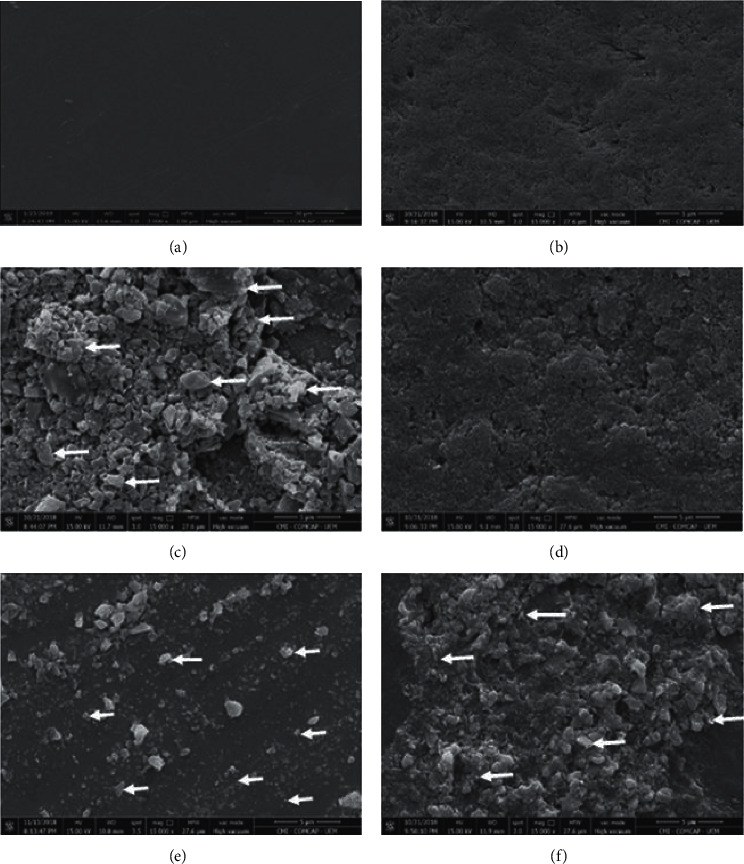
Representative scanning electron micrographs of the enamel surface morphology. The arrows indicate the presence of precipitates; (a) intact enamel—IE: is observed a smooth surface due to sequential polishing; (b) demineralized enamel—DE: disorganized enamel surface is observed; (c) silver diamine fluoride—SDF: presence of a layer with agglomerated precipitates (arrow) and heterogeneous on demineralized enamel surface treated with SDF; (d) fluoride—F: can be observed a disorganized enamel surface; (e) silver—Ag: scattered areas of enamel precipitates (arrow); (f) silver nanoparticles and fluoride—AgF: presence of a layer with precipitates (arrow) agglomerated and heterogeneous on the surface of decayed enamel treated with AgF.

Figure 5 shows the SEM images of the enamel surface. In the qualitative analysis of enamel surfaces, SEM images allowed observing the groups treated with different solutions (SDF, AgF, and Ag) presented some degree of dental enamel precipitation different from DE group, which presented the surface completely disorganized (Figure 5(b)) and IE (Figure 5(a)), that is intact). The presence of P, Ca, Au, Ag, and Cl was observed by EDS under SEM, especially in the SDF, Ag, and AgF.

#### 3.3.4. Photographic Images

**Figure 6:**
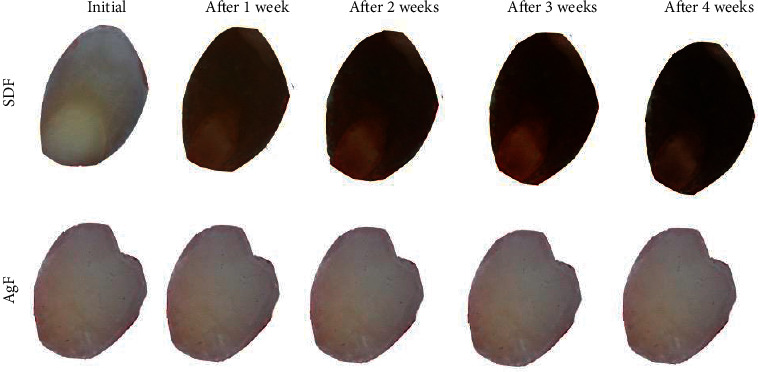
Photographic image of superficial enamel demineralized specimens treated with SDF and AgF, 4-week follow-up.

**Figure 7:**
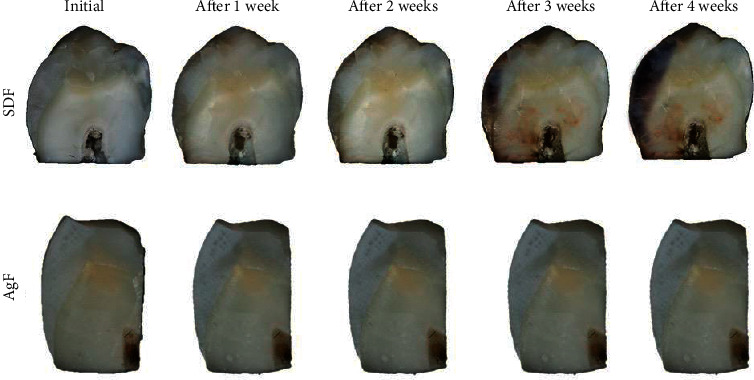
Photographic image of cross-sectional enamel demineralized specimens treated with SDF and AgF, 4-week follow-up.

Figures 6 and 7 show the enamel superficial and cross-sectional color changes by SDF and AgF for 4 weeks follow-up. The images revealed that all specimens treated with SDF exhibited some degree of increasing staining on both the surface and the internal enamel area since the first week. However, the samples treated with AgF did not show visible color.

## 4. Discussion

This study carried out the physical and biological characterization of an experimental anticaries agent based on silver nanoparticles and fluoride (AgF). It revealed that the application of AgF had a similar effect to the SDF on enamel remineralizing with antimicrobial effect without cytotoxicity. The null hypothesis that no difference exists in the effects between the anticaries agents tested (SDF and AgF) cannot be rejected.

The antimicrobial tests showed that AgF was able to inactivate 100% growth of *S. mutans*, *E. faecalis*, and *E. coli* at a lower concentration of active compounds than SDF and CHX (a positive control). This result may be related to the nanometric size of the silver particles used in the experimental solution (7–30 nm). Nanometric particles have a larger surface area available to interact with microorganisms [2, 11–13, 16], and they have different action mechanisms than ionic silver and become more potent than bigger particles and ionic silver [9, 13]. Consequently, lower concentrations are needed to get the antimicrobial in action [9, 16].

The MBC value found was corresponding to the MIC: 160 µg/mL, which is different from the results of other studies with experimental solutions [13]. The difference between results can occur because of the need for different concentrations of silver nanoparticles to obtain antimicrobial action on the same type of microorganism. It is known that differences in nanoparticle sizes [9, 12] and formats can directly influence the antimicrobial effect [16]. It is possible that nanoparticles used in other studies had different morphological characteristics than the ones used in this study.

The absence of microbial activity in the ADT test is indicated by the value 1.0 (*a* = *b*), so values equal to or greater than 1 indicate that the solution had antimicrobial activity. In this study, all tested solutions presented inhibition zones. For *S. mutans* and *E. faecalis*, SDF showed the largest inhibition halo, followed by CHX, which was larger than AgF and Ag. For *E. coli*, SDF presented a larger zone and was statistically different from the other solutions tested. AgF was the unique solution that presented a statistical difference between the three microorganisms. Those results were similar to the study by Schwass et al. [13] that some microorganisms are more resistant than others, which justify that AgF results are different in these microorganisms. Although the values obtained by AgF and Ag are lower than those of the reference solutions (SDF and CHX), they are equivalent to 100% inhibition of microbial activity, because they were greater than 1.0, which corresponds to the absence of microbial activity, results that confirm those obtained in MIC. These results suggest that the antimicrobial action is rationed to silver nanoparticles; thus, it is possible to observe that the addition of fluoride to the experimental solution did not statistically increase this effect.

In the cytotoxicity test, AgF and SDF presented similar cellular viability, causing little damage to the cells. Other experimental solutions with silver nanoparticles in low concentrations were previously tested [9, 16] and had no cytotoxicity in human cells [25]. The silver nanoparticles' biocompatibility to mammals' cells was verified, and the use of it in dental materials was suggested to not cause a threat to human health [26, 27]. However, more studies are needed to determine the best concentration of silver nanoparticles that can ensure antimicrobial action without the increase of cytotoxicity [26, 27]. The comparison of biological results with other studies is very complex because of the specific characteristics of the different bacterial strains [9, 13] of the stabilizing agents used in the experimental formulation and silver nanoparticles size [9, 12].

For silver nanoparticles to be sufficiently effective and to replace ionic silver in anticaries agents, they need to remain dispersed in a colloidal solution. This dispersion characterizes the stability of the solution and guarantees the antimicrobial and remineralizing effects [13]. Also, the presence of fluoride is necessary to potentiate such effects [4, 8, 14]; however, there is difficulty in stabilizing silver nanoparticles in contact with fluoride [28].

The micrograph images obtained in TEM demonstrate the dispersion of the nanoparticles, which provides the stability of the experimental formulation, shortly after preparing the solution (Figure 2(a)) also in the long term, 12 months (Figure 2(c)). In this study, the stability of a solution containing silver nanoparticles and fluoride for 12 months makes this solution clinically applicable, which was not demonstrated in other studies [8, 9, 16, 28].

The colloidal silver nanoparticles solution was obtained through chemical coprecipitation reactions with pH from 3 to 5 involving the simultaneous production and dispersion of the nanoparticles into the base fluid. Ethylene glycol was used as a solvent [29] with water as a vehicle. A polyvinylpyrrolidone (PVP) treatment was applied to silver nanoparticles as reducing agent because it influences the aspect ratio of the nanoparticles [25] and ensures good solution stability. Silver nanoparticles coated with PVP dispersed in colloidal solution keep the nanoparticles in suspension and distant from each other [30]. In these ways, stability in colloidal form was got without silver nanoparticles aggregating and precipitating, keeping the unique enhanced properties associated with being nanosized maintain product shelf life and for substantive antimicrobial effects desired for dental application [13].

Besides, it was possible to characterize the silver nanoparticles with a predominant spherical shape and an approximate size of 7–30 nm, size equivalent to those described in previous studies that found bactericidal and bacteriostatic effects of solutions with silver nanoparticles measuring between 5 and 98 nm, against *S. mutans* of the ATCC [9, 13].

After evaluating the AgF stability and antimicrobial action without cytotoxicity, we assessed the possible remineralizing action on human dental enamel. Samples of human dental enamel were used to get results as close as possible to clinical ones, despite being an *in vitro* study. A pH-cycling protocol was used to obtain the initial caries lesion; this is to simulate imbalance of the des-remineralization process that triggers dental caries *in vivo* situations [28].

When evaluating the superficial microhardness of the demineralized dental enamel treated with SDF, AgF, Ag, and F, a higher percentage of superficial microhardness alteration was verified on the surfaces treated with SDF and AgF. These results showed that the fluoridated solution based on silver nanoparticles presented remineralizing action similar to SDF. This action may be the result of a synergistic effect from the association of silver nanoparticles and fluoride. In view that the effect obtained by the association of silver nanoparticles and fluoride was superior to that obtained by the isolated application of each of these solution components, a result also observed in previous studies [4, 8, 14, 16]. dos Santos et al. [8], in a randomized clinical trial, verified the effectiveness of an experimental fluoridated solution based on silver nanoparticles to prevent dental enamel caries and stated that this effect can be explained by the synergism of formulation components. The same synergy was observed by Nozari et al. [14], in an *in vitro* study with human deciduous teeth, in which they found that the highest values of remineralization obtained came from the association of silver nanoparticles and fluoride. Thus, the action of silver nanoparticles can be enhanced by their combination with fluoride [16], and both are responsible for the remineralization of dental enamel [14].

The alteration in mineral volume was also evaluated in depth (up to 200 *μ*m) to verify the possible remineralizing action promoted by AgF, as well as on the surface. The AgF group showed a greater gain in mineral volume at all depths, and its application on demineralized enamel surface resulted in a higher mineral volume than the intact enamel (Figure 4). This result was also seen in the group that received SDF, in some depths. Zhao et al. [5], after a systematic review of the literature, stated that the SDF application on demineralized enamel surfaces significantly increased the microhardness at a depth ±150 *μ*m and that levels of calcium and phosphorus increased from the surface to the depth 300 *μ*m. This in-depth remineralization may indicate that solutions have penetrated beyond the enamel surface.

The remineralization obtained in this study differs from the study of Scarpelli et al. [28], in which they evaluated a silver nanoparticle experimental solution remineralizing effect on the deciduous human enamel surface and in depth. When comparing the action of the experimental solution to SDF, the experimental solution got the lower remineralizing performance. However, their solution had only silver nanoparticles and no fluoride in composition, because of the difficulty to stabilize nanoparticles in contact with fluoride, as reported by the authors [28]. The solution of silver ions could infiltrate carious lesions, precipitate, and result in increased dental enamel microhardness [14]. However, the absence of fluoride in the composition tested may be responsible for the lower performance of the experimental solution, which did not occur in the present study because AgF has in its composition both silver nanoparticles and fluoride.

A precipitate layer was observed in the SDF group in SEM image (Figure 5(c)), resulting from the product reaction with hydroxyapatite's demineralized surface, consequent formation of calcium fluoride (CaF_2_), and silver phosphate (Ag_3_PO_4_) [14]. Other studies [5, 14] reported the formation of a dense and highly remineralized surface layer, resulting from the SDF application on the decayed surface. This layer is rich in calcium and phosphate and can directly reflect the clinical situation in which the surface of the lesion interrupted by the SDF action becomes hardened. The mineral composition analysis of the region (EDS) confirmed the presence of calcium, phosphorus, and silver but did not detect the fluoride presence. The absence of fluoride may have occurred because the residual fluoride in the samples could be below the EDS detection limit; this is because calcium fluoride is more soluble than hydroxyapatite [5] and dissociates into calcium and fluoride ions [5]. Fluoride ions are absorbed by apatite crystals and replace hydroxyl ions in the crystal; this fluoride absorption is accompanied by an increase in the size of the apatite crystal [4, 31]. In the AgF group, the presence of an irregular precipitated layer was also observed. In the Ag and F groups, it did not happen, and there were some precipitates, but these were found less concentrated and dispersed without a remineralization layer. These findings reaffirmed the results microhardness surface alteration, in which the synergistic effect of silver nanoparticles and fluoride was observed.

In addition to phosphate and calcium, silver chloride is also one of the possible products by silver reaction during the remineralization process and can be detected on the surface by EDS [4, 5, 31]. Silver chloride may be present on the surface during remineralization [5], and this could explain the detection the chlorine presence (EDS) in some of the groups that showed precipitate formation on the enamel surface (Figures 5(c), 5(e), and 5(f)). Silver chloride is the product most found in precipitates, resulting from the SDF application, according to Yu et al. [31].

The photographic images of demineralized samples treated with SDF and AgF allowed us to verify the change in the enamel color in those treated with SDF both on the enamel surface and in depth, whereas those treated with AgF did not show visible dental tissue color change (Figures 6 and 7). This coloring was the result of SDF's silver particle precipitation, forming a silver phosphate layer in the demineralized enamel, followed by oxidation of particles in which time was the determining factor for the extent of enamel coloring [10, 15]. It is possible to observe through the images that over time, the blackening of the enamel became more evident, and in the fourth week, the enamel became darker. These findings corroborate with some studies that demonstrated the silver nanoparticle anticaries agents can arrest the caries progress without staining the dental surface [8, 9, 15]. The enamel treated with AgF was not staining, which can be justified by the presence of silver nanoparticles, and which does not form oxides in contact with oxygen in the medium, and thus not causing color changes in the tooth enamel [15].

It is important to highlight that this study evaluated the effect of cariostatic agents on in vitro conditions, the absence of in vivo conditions such as salivary enzyme attacks, continuous changes in pH, and oral cavity temperature might mitigate the effects on enamel remineralization. Thus, future studies evaluating the effect of these substances in situ and in vivo should be carried out to confirm these findings.

## 5. Conclusion

The silver nanoparticles and fluoride demonstrated antimicrobial potential and biocompatibility, and superficial and in-depth remineralizing capacity with long-term solution stability and, furthermore, presented similar results to silver diamine fluoride, without darkening the enamel surface.

## Figures and Tables

**Table 1:** Median inhibition zone description (mm) of SDF, CHX, AgF, and Ag.

Treatment	Microorganism		
	*S. mutans*	*E. faecalis*	*E. coli*
SDF	5.94^Aa^	3.62^Ab^	3.68^Ab^
CHX	5.11^Ba^	2.15^Bb^	2.05^Bb^
AgF	2.67^Ca^	1.40^Cc^	2.05^Bb^
Ag	2.66^Ca^	1.63^Cb^	1.99^Bb^

Medians followed by distinct letters (lowercase letters in row; uppercase letters in columns) are significantly different by Dunn's test (*p* < 0.05).

**Table 2:** Mean values (standard deviation) of the initial surface microhardness (SM_*i*_), after pH-cycling microhardness (SM_pH_), final surface microhardness (SM_*f*_), percentage of surface remineralization (%SM), and variation of microhardness (ΔSM), *n* = 12.

Experimental group	SM_*i*_	SM_pH_	SM_*f*_	%SM	ΔSM
SDF	324.6 (32.7)^Aa^	150.6 (27.8)^Ac^	222.6 (34.8)^Ab^	43.5 (18.4)^A^	71.9 (28.7)^A^
F	324.1 (38.7)^Aa^	131.8 (33.8)^Ab^	137.7 (31.8)^Bb^	3.1 (4.1)^B^	5.8 (7.8)^B^
AgF	336.7 (47.6)^Aa^	146.8 (31.1)^Ac^	217.6 (44.1)^Ab^	43.6 (26.3)^A^	70.7 (26.9)^A^
Ag	334.1 (30.3)^Aa^	149.2 (27.8)^Ab^	155.4 (31.8)^Bb^	3.8 (8.2)^B^	6.2 (12.9)^B^

Means followed by different lowercase letters, in line, differ statistically by Tukey's test; means followed by different uppercase letters, in column, differ statistically by Tukey's test. The %SH and DSM were compared only between the experimental groups.

## Data Availability

The data used to support the findings of this study are available from the corresponding author upon request.
